# Gastric pH and serum gastrin concentration in age‐matched healthy dogs and dogs with chronic kidney disease

**DOI:** 10.1111/jvim.16907

**Published:** 2023-10-24

**Authors:** Kylie Grady, Eli Ernst, Patricia L. Secoura, Josh Price, Adam Birkenheuer, Shelly L. Vaden, Jonathan Lidbury, Emily Gould, Joerg M. Steiner, M. Katherine Tolbert

**Affiliations:** ^1^ Department of Molecular Biomedical Sciences College of Veterinary Medicine, North Carolina State University Raleigh North Carolina USA; ^2^ Department of Small Animal Clinical Sciences, College of Veterinary Medicine University of Tennessee Knoxville Tennessee USA; ^3^ Department of Small Animal Clinical Sciences, College of Veterinary Medicine and Biomedical Sciences Texas A&M University College Station Texas USA; ^4^ Present address: Care Center Dayton Ohio USA

**Keywords:** acid suppressant, azotemia, canine, famotidine, omeprazole

## Abstract

**Background:**

Gastric hyperacidity and hypergastrinemia are purported to cause gastric ulceration in dogs with chronic kidney disease (CKD); however, no published studies have evaluated gastric pH with serum gastrin concentrations in dogs with CKD.

**Hypothesis:**

To compare mean intragastric pH, mean percent pH distribution, and serum gastrin concentrations in dogs with CKD to age‐matched, healthy dogs. We hypothesized there would be no difference in mean gastric pH or serum gastrin between groups.

**Animals:**

Thirteen dogs with CKD; 10 aged‐matched healthy dogs.

**Methods:**

Prospective, case‐control study. Serum chemistry, complete blood count, urinalysis, and serum gastrin concentrations were evaluated in all dogs before radiographic‐assisted gastric placement of a pH capsule. Forty‐eight‐hour continuous gastric pH monitoring was performed in all dogs. Serum gastrin concentration, mean pH, and mean percentage time that gastric pH was strongly acidic (pH <1 and pH <2) were compared between groups using a repeated measures mixed‐model ANOVA.

**Results:**

No significant differences were observed between groups for any pH measurements, including mean ± SD gastric pH (CKD, 2.37 ± 0.87; healthy, 2.39 ± 0.99; *P* > .05). Serum gastrin concentrations were not significantly different between groups (median [range]: CKD, 10.5 ng/dL [<10‐17.1]; healthy, 10.9 ng/dL [<10‐15]; *P* > .05).

**Conclusions and Clinical Importance:**

Our client‐owned dogs with CKD did not have lower gastric pH or higher serum gastrin concentrations compared to healthy dogs. Our results suggest that prophylactic gastric acid suppression in dogs with CKD is not warranted unless other clinical indications for use are present.

**Abbreviations**
CKD: chronic kidney disease
GI: gastrointestinal
IRIS: International Renal Interest Society
MPT: mean percentage time

## 1 INTRODUCTION

Chronic kidney disease (CKD) can be a progressive, irreversible disease estimated to affect up to 25% of dogs.^1^ Animals with advanced disease frequently develop uremic gastritis or gastrointestinal (GI) signs, such as vomiting or anorexia, that can affect healthspan.^2^, ^3^ Historically, CKD‐induced GI signs have been attributed to higher circulating uremic toxins and decreased renal clearance of gastrin, a hormone that in excess, can cause gastric hyperacidity and ulceration.^3^, ^4^, ^5^ In humans with end‐stage kidney disease, acid‐related upper GI diseases, such as peptic ulcers and erosive gastroduodenitis, are common, and these patients frequently receive acid suppressants.^6^, ^7^, ^8^

Prophylactic acid suppression is recommended for dogs and cats with CKD^3^, ^9^; however, there is limited to no evidence to support its use. Mean gastric pH is similar between healthy, age‐matched cats and those with CKD,^10^ and while some studies report higher serum gastrin concentrations in cats with CKD compared to healthy cats,^4^, ^5^ others report no difference.^10^ In addition, unlike humans, cats with CKD develop gastric fibrosis and mineralization as opposed to erosive or ulcerative lesions.^4^ In dogs, the relationship between CKD, gastric hyperacidity, and GI ulceration is unknown. Gastroduodenal ulceration and erosion are not commonly associated with kidney disease in dogs^11^; rather, primary lesions identified in dogs with uremic gastropathy include gastric edema, mineralization, and vasculopathy.^2^ However, ulceration is noted in a small number of dogs with kidney disease,^2^, ^12^, ^13^ and dogs with CKD have a higher incidence of fecal occult blood positivity.^14^ No studies have evaluated gastric pH or serum gastrin concentrations in dogs with CKD, and thus, appropriate treatment recommendations for these dogs are unknown.

Despite this lack of information, empiric acid suppression is common among animals with CKD^15^ but might not be benign. Chronic proton pump inhibitor use is associated with a higher risk of fractures^16^ and accelerated bone mineral loss in humans.^17^ This could present serious risk to a large number of dogs with CKD with renal mineral and bone disorders (>75%) which already suffer from decreased bone quality.^18^, ^19^

Consequently, given that no studies have evaluated if acid suppression is indicated in dogs with CKD, this study had 2 objectives. The first objective was to evaluate mean intragastric pH and mean percent pH distribution in dogs with CKD compared to age‐matched, healthy dogs. The second objective was to determine if serum gastrin is higher in dogs with naturally occurring CKD. We hypothesized that there would be no significant difference in serum gastrin concentrations or pH measurements between healthy dogs and dogs with CKD.

## 2 METHODS

### 2.1 Study animals

The Institutional Animal Care and Use Committee (IACUC) at Texas A&M University, North Carolina State University, and University of Tennessee approved the protocols for this study. To detect a hypothesized paired mean difference in mean pH of 0.4 between dogs with CKD and healthy dogs, assuming a SD of 0.4096 for each group, a correlation of 0.5, at an alpha of 0.05, 11 pairs of dogs were required per group to achieve 80% power. Client‐owned healthy adult dogs and dogs with stable CKD were prospectively enrolled in the study from September 2019 to September 2021 from 3 veterinary university hospitals (Texas A&M University, North Carolina State University, and University of Tennessee). One affected male laboratory dog with an X‐linked hereditary nephropathy was also enrolled at Texas A&M University. All owners completed a consent form before study enrollment. Inclusion criteria for dogs with CKD included a body weight >3.0 kg, Stage I to IV CKD as defined by the International Renal Interest Society (IRIS), and USG <1.015. Inclusion criteria for healthy control dogs included body weight >3.0 kg, lack of azotemia (International Renal Interest Society. IRIS Staging of CKD; 2023. http://www.iriskidney.com/pdf/2_IRIS_Staging_of_CKD_2023.pdf; ie serum creatinine <1.4), USG >1.015, a serum symmetric dimethylarginine (SDMA) within reference range (<14 μg/dL), a body condition ≥4 (out of 9; WSAVA Global Nutrition Committee. Body condition score; 2013. https://wsava.org/wp-content/uploads/2020/01/Body-Condition-Score-Dog.pdf), and no recent (<2 months) history of medication administration other than routine heartworm and flea prophylaxis. The requirement for healthy dogs having an SDMA <14 μg/dL was added following case screening and initial inclusion. Exclusion criteria in either group included the presence of other systemic diseases (eg, diabetes mellitus, hepatitis, history, or evidence of chronic primary GI disease) or the use of any other drug that might alter intragastric pH within the past 7 days before or during intragastric pH monitoring (eg, NSAIDS, steroids, antibiotics).

Given that serum gastrin concentration normalizes after a 7‐day discontinuation of omeprazole and famotidine,^20^ acid suppressants were discontinued for a minimum of 8 days before and during pH monitoring. Oral administration of aluminum hydroxide was stopped for 48 hours before and during pH monitoring due to their neutralizing effect on gastric acid. This is well beyond the likely duration of action of aluminum hydroxide, which has a duration in humans of approximately 26 minutes.^21^ As the majority of dogs with CKD were eating a renal therapeutic diet, healthy dogs were also fed a therapeutic renal diet (Royal Canin Renal Support A, Royal Canin USA Inc., St. Charles, Missouri) 3 to 5 days before the study and during pH monitoring to minimize the effect of diet as a confounding variable. All dogs had a complete blood count, serum biochemistry, urinalysis, urine culture, serum gastrin, and blood pressure measurement within 72 hours of pH monitoring. Muscle condition score was recorded for all animals (WSAVA Global Nutrition Committee. Muscle Condition Score. 2013; https://wsava.org/wp306content/uploads/2020/01/Muscle-Condition-Score-Chart-for-Dogs.pdf). Dog's IRIS stage was determined by serum creatinine. All blood tests, with the exception of serum gastrin, were performed by the respective institutional clinical pathology service. For measurement of serum gastrin concentrations, serum was collected from blood tubes after centrifugation at 250*g* and stored in cryovials at −80°C. Serum was shipped on dry ice to the Gastrointestinal Laboratory at Texas A&M University. Serum gastrin concentrations were measured by an automated chemiluminescence, enzyme‐labeled immunometric assay (Immulite 2000, Siemens Healthcare Diagnostics, Malvern, Pennsylvania), as previously described.^22^

### 2.2 pH capsules

All dogs were lightly sedated with butorphanol (0.3 mg/kg IV; Torbugesic 10 mg/mL injection; Fort Dodge Animal Health, Fort Dodge, Iowa) and dexmedetomidine (1 mcg/kg IV [CKD] or 2 mcg/kg IV [healthy]; Dexdomitor 0.5 mg/mL injection; Orion Pharma, Espoo, Finland). A Bravo pH calibration free capsule (Medtronic, Minneapolis, Minnesota) was passed orally using a delivery device attached to the capsule. Once positioning of the capsule in the stomach was confirmed radiographically, mucosal attachment of the pH capsule was achieved as previously reported.^23^ Before use, all pH capsules and receivers were linked as previously described according to the manufacturer's instructions.^24^ After the procedure, the sedation was reversed with an equal volume of atipamezole (0.05‐0.1 mg/kg IM; Antisedan 5 mg/mL injection; Orion Pharma, Espoo, Finland). Dogs were allowed to eat once fully recovered and in the care of the clients. Neither the amount of food fed nor the timing of feeding was standardized. Clients of dogs with CKD were instructed to feed as they previously had, and clients of healthy dogs were given specific amounts to feed of the renal diet that was roughly equivalent to what they have been feeding with the dog's maintenance diet. Gastric pH recordings were obtained telemetrically at 6‐second sampling intervals for 48 hours after pH capsule administration. Receivers were maintained in a vest worn by the dog during the 48‐hour monitoring period. The pH data were uploaded to the computer by proprietary software (Reflux Software v6.1, Medtronic) after the monitoring period. Early capsule detachment from the stomach was defined by a rapid and persistent rise in pH >4. Only data from the first 48 hours of recording in the stomach were included in the analysis. Mean pH and mean percentage of time (MPT) that gastric pH was 0 to 1 and 1 to 2 were calculated using data from the proprietary software supplied by the device manufacturer.

### 2.3 Statistical analysis

A longitudinal matched case‐control study design and corresponding mixed model analysis of variance (ANOVA) was performed to evaluate mean intragastric pH and MPT that intragastric pH was ≤1 and ≤2. Each outcome was evaluated for status (CKD/healthy), time (day 1/day 2), and status‐by‐time differences. Dogs were individually paired based on the closest similar age of dogs with CKD and healthy dogs. Each matched pair, and status nested within pair were considered random effects.^25^ A Shapiro‐Wilk test and QQ plots were used to evaluate normality of ANOVA residuals for each outcome. Levene's equality of variances test was used to evaluate equality of treatment variances. Box‐and‐whisker plots and studentized residual diagnostics were performed to evaluate each mixed model for the presence of outliers. A log transformation of MPT ≤1 was required to meet underlying statistical assumptions. After applying a log transformation for MPT ≤1, all statistical assumptions were met.

Both 48‐hour mean gastric pH and serum creatinine measures were evaluated for normality using the Shapiro‐Wilk Test. Since healthy dog creatinine was not normally distributed, Spearman's correlations analysis was performed to evaluate whether a significant relationship was present between pH and creatinine for both CKD and healthy dogs.

Serum gastrin values below the detectable limit were conservatively assumed to be at the limit of detection of 10 ng/dL. Serum gastrin concentration differences were evaluated between age‐matched CKD and healthy dogs using a Shapiro‐Wilk test and determined to not be normally distributed. Subsequently, a Wilcoxon Signed Rank Test was performed to evaluate serum gastrin for mean rank status differences. Statistical significance was defined as *P* < .05. Statistical analysis was performed using commercial software (SAS software, version 9.4, Cary, North Carolina, Release TS1M7 and IBM SPSS Statistics software, Version 29, Armonk, New York).

## 3 RESULTS

### 3.1 Dogs

Thirteen dogs with CKD and 11 healthy dogs were enrolled. One healthy dog was determined to have an SDMA (18 μg/dL) outside of the reference range following inclusion and was subsequently excluded. One dog with CKD underwent pH monitoring and passed the capsule after 24 hours; a second capsule was not administered and only the first 24 hours were included in the analysis. Demographic information is summarized in Table 1, including number of dogs per group, breeds, age, sex, weight, body condition score, and muscle condition score. There was no significant difference in age between groups, and 2 dogs with CKD did not have a muscle condition score recorded.

**TABLE 1:** Demographic information of enrolled study dogs. Muscle condition score with 3 being normal muscling, 2 being mild muscle loss, 1 being moderate muscle loss, and 0 being severe muscle loss.

Group	Number of dogs	Sex	Mean age, years (SD)	Median weight, kilograms (range)	Median body condition score out of 9 (range)	Median muscle condition score out of 3 (range)	Breeds
CKD	13	1F, 4 NM, 8 SF	8.7 (5.4)	19.3 (3.4‐32.7)	4 (3‐7)	2 (1‐3)	Beagle (n = 1), Boxer (n = 1), Chow Chow (n = 1), Golden Retriever (n = 1), Labrador Retriever (n = 3), Mixed breed (n = 6)
Healthy	10	3 NM, 7 SF	8.6 (3.4)	20 (9.9‐38.2)	5 (4‐6)	3 (2‐3)	Australian Cattle dog (n = 1), Beagle (n = 1), Huskey (n = 1), Labrador Retriever (n = 1), Mixed breed (n = 5), Shetland Sheepdog (n = 1)

Abbreviations: F, intact female; NM, neutered male; SF, spayed female.

In total, 3 dogs with IRIS stage I, 3 dogs with IRIS stage II, 2 dogs with IRIS stage III, and 5 dogs with IRIS stage IV CKD were enrolled. All healthy dogs had hematocrits within the reference range. Seven out of 13 dogs with CKD were anemic (median, range hematocrit: 35, 33‐50; reference interval: 40‐61). Out of these 7 dogs, 2 were microcytic and 2 were macrocytic.

Dogs with CKD were receiving the following medications or treatments for CKD: anti‐proteinuric or anti‐hypertensive medications (telmisartan, n = 2 or enalapril, n = 1 or amlodipine, n = 1), as needed maropitant (n = 2), as needed mirtazapine (n = 1), calcitriol (n = 2), daily subcutaneous fluids (n = 1), and phosphate binders (n = 3; all aluminum hydroxide). Three dogs were receiving acid suppressants (omeprazole n = 2, famotidine n = 1) before discontinuation for study enrollment. Eleven dogs with CKD were eating a therapeutic renal diet at the time of pH monitoring (Hill's k/d, Hill's Pet Nutrition, Inc., Topeka, KS [n = 6]; Royal Canin Renal, Royal Canin USA Inc., St. Charles, Missouri [n = 4]; Royal Canin Feline Renal, Royal Canin USA Inc., St. Charles, Missouri [n = 1]; Blue Buffalo Kidney Support, Blue Buffalo Co., Wilton, Connecticut [n = 1]; home‐prepared diet [n = 1]; laboratory chow diet [n = 1]). All healthy dogs accepted and were fed the therapeutic renal diet before and during the study period.

### 3.2 pH monitoring and serum gastrin

**FIGURE 1:**
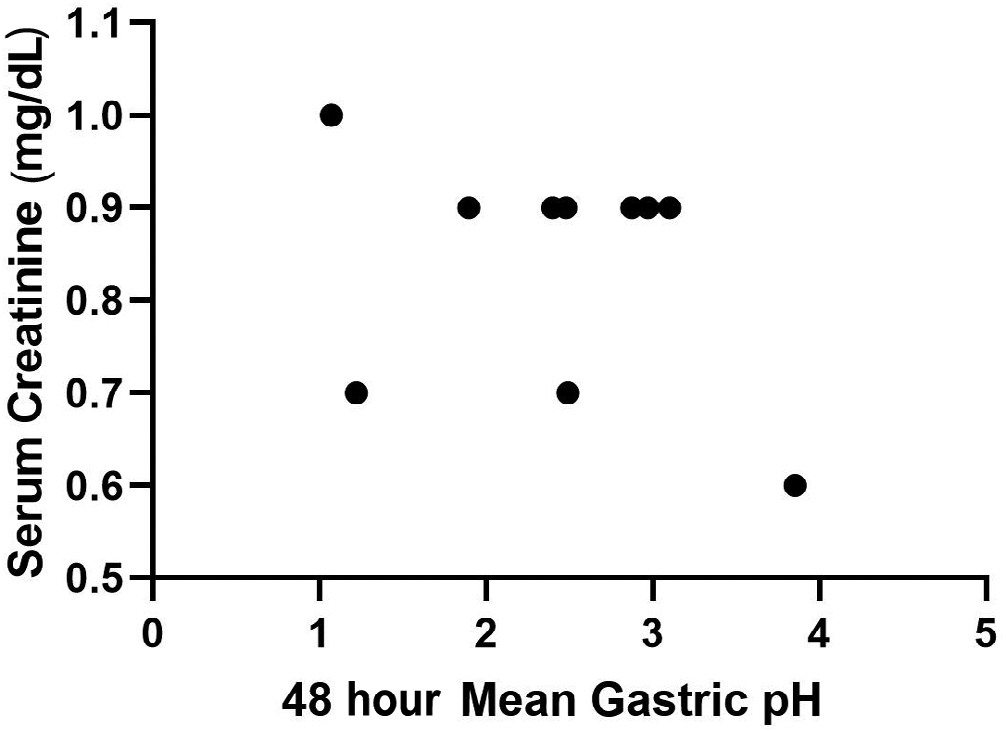
Scatterplot of 48‐hour mean gastric pH and serum creatinine in healthy dogs. No correlation between 48‐hour mean gastric pH and serum creatinine was noted in healthy dogs (*ρ* = −0.39, *P* = .26).

**FIGURE 2:**
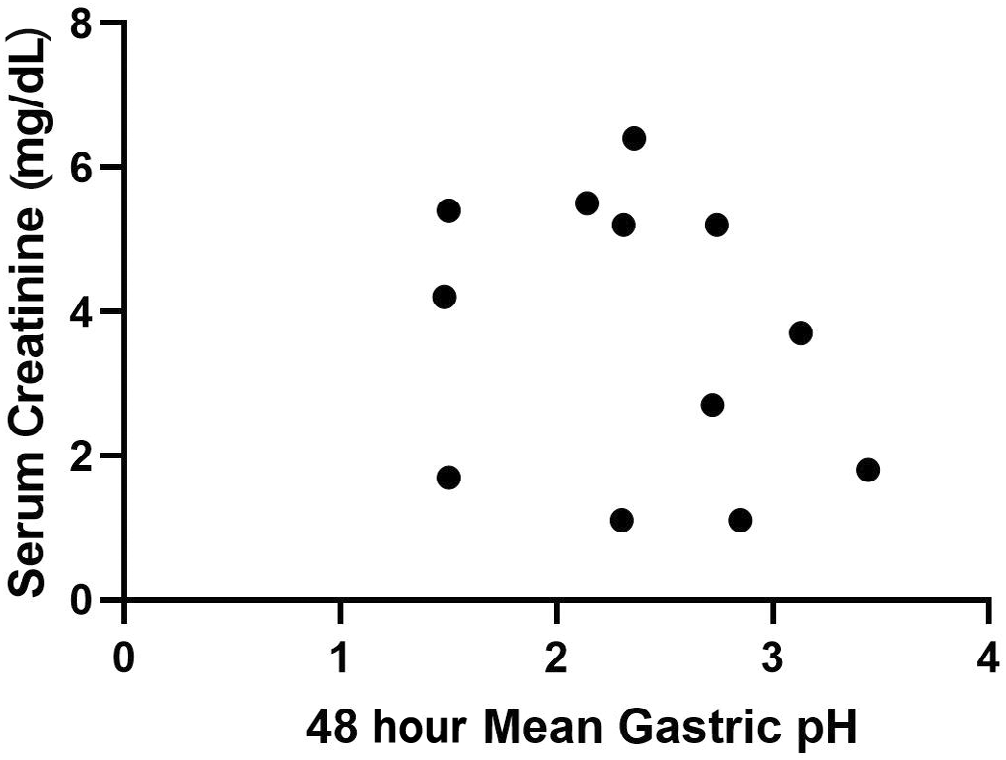
Scatterplot of 48‐hour mean gastric pH and serum creatinine in dogs with chronic kidney disease. No correlation between 48‐hour mean gastric pH and serum creatinine was noted in dogs with chronic kidney disease (*ρ* = −.25, *P* = .43).

Mean gastric pH and the percentage of time that gastric pH was strongly acidic (pH <1 and <2) during day 1 (first 24‐hour period) and day 2 (second 24‐hour period) were used for comparative analyses and are presented in Table 2. No significant difference was observed in status (CKD/healthy), time (day 1/day 2), or the status by day interaction for mean pH, or mean percentage time that gastric pH was strongly acidic (*P* > .05, for all). Spearman's correlation for serum creatinine and gastric pH for both the CKD (*ρ* = −0.25, *P* = .43) and healthy (*ρ* = −0.39, *P* = .26) dogs were not significant (Figures 1 and 2).

**TABLE 2:** Gastric pH values and mean percentage of time (MPT) pH were strongly acidic (<1 or <2) in healthy dogs and dogs with chronic kidney disease (CKD) over time.

Health status	Day	Variable	Mean (SD)	Range
CKD	1	Mean pH	2.6 (0.9)	1.3‐4.2
		MPT pH <1	13.3 (17.7)	0.02‐64.0
		MPT pH <2	51.1 (25.2)	7.8‐88.1
	2	Mean pH	2.1 (0.8)	0.73‐3.6
		MPT pH <1	13.7 (15.0)	3.3‐52.1
		MPT pH <2	61.0 (16.6)	35.3‐86.6
Healthy	1	Mean pH	2.5 (0.9)	1.14‐4.4
		MPT pH <1	10.6 (14.5)	0.2‐48.8
		MPT pH <2	53.4 (24.2)	25.6‐93.2
	2	Mean pH	2.3 (1.1)	0.8‐4.0
		MPT pH <1	24.2 (33.9)	0.1‐94.0
		MPT pH <2	60.8 (25.8)	34.4‐99.9

All dogs with CKD (median [range], 10.5 ng/dL [<10‐17.1]) and healthy dogs (median [range], 10.9 ng/dL [<10‐15]) had serum gastrin concentrations within the reference range. Two dogs with CKD did not have gastrin data available. Serum gastrin concentrations were not significantly different between CKD and healthy dogs (*P* > .05).

## 4 DISCUSSION

In this prospective, case‐controlled study, we evaluated gastric pH and serum gastrin concentrations in dogs with CKD. In our study sample, dogs with CKD and healthy dogs had no significant difference in mean gastric pH, serum gastrin concentration, or mean percentage time that gastric pH was strongly acidic. These findings do not support prophylactic gastric acid suppression in dogs with CKD unless other risk factors for GI bleeding are present.

Historically, prophylactic gastric acid suppression was recommended for the treatment of dogs with CKD and GI signs^3^, ^9^ due to the concern that decreased renal clearance of the gastric acid secretagogue, gastrin, would result in gastric hyperacidity and ulceration.^4^, ^5^ However, growing research suggests that dogs and cats with CKD, especially earlier stages, might not experience significant hypergastrinemia and gastric hyperacidity. While 2 earlier studies demonstrated higher gastrin concentrations in cats with CKD compared to healthy cats,^4^, ^5^ neither study controlled for age, which might impact gastrin concentrations based on work performed in humans.^26^ In addition, in both studies, at least 70% of cats with CKD had moderate to severe kidney disease, limiting assessment of gastrin concentrations in cats with earlier stage CKD. In contrast, 1 small age‐controlled study of 19 cats found no significant difference in gastrin or gastric pH between cats with or without CKD,^10^ and across all studies, serum gastrin concentrations were highly variable in cats with CKD. Our study also found no significant difference in mean gastric pH or serum gastrin concentrations in dogs with CKD versus age‐matched healthy controls. Together, these findings support the current consensus that use of gastroprotectants in dogs and cats with IRIS stages I to III CKD is not indicated in the absence of evidence of GI bleeding.^27^ In dogs with CKD and GI signs such as vomiting or dysorexia, anti‐nausea or anti‐emetic medications should be considered first, as other factors, such as increased uremic toxins or centrally acting emetogens, are more likely causes of those clinical signs.

While this study did not support hyperacidity or hypergastrinemia in dogs with CKD, the prevalence of gastroduodenal ulceration in this population is unknown. Dogs with kidney failure are more likely to have evidence of gastric edema, mineralization, and vasculopathy on postmortem histopathology, and histologic ulceration is infrequently observed.^2^ However, some studies do report rare ulceration in dogs with CKD,^2^, ^12^, ^13^ and while 1 study found that dogs with CKD had a significantly higher incidence of fecal occult blood,^14^ it did not control for diet, which can impact fecal occult blood tests. Ultimately, further research is needed to determine both the prevalence and pathogenesis of GI ulceration in dogs with CKD. Based on this study, gastric hyperacidity is likely not the cause, and other abnormalities observed in dogs with CKD, such as altered gastric surface mucous and gastric microvascular changes, should be considered instead.^2^, ^12^, ^13^ In the meantime, acid suppression should be restricted to dogs with CKD who either have additional risk factors for ulceration or have evidence of GI bleeding (hematemesis, melena, iron‐deficiency anemia).^27^ Among our sample of dogs, only 3 out of 13 dogs with CKD had hematologic or biochemical changes suggestive of a GI bleed, including a microcytic anemia or a discordant BUN : creatinine ratio.^28^ If acid suppression is started in dogs with a suspicion for a GI bleed, routine monitoring for resolution of clinical signs or bloodwork abnormalities should be pursued. Acid suppression should be stopped if no improvement is noted.

One limitation of this study was that a small group of dogs was evaluated and only one‐half of the dogs had advanced disease (ie, IRIS Stages III‐IV). While inclusion of more dogs in later stages might have resulted in a significant difference in gastric pH or serum gastrin, no relationship was observed between serum creatinine and 48‐hour mean gastric pH. Moreover, our use of a urine‐specific gravity of >1.015 to exclude renal disease in our healthy control group could have resulted in some dogs with early kidney disease being included; however, our later addition to exclude dogs with an SDMA ≥14 μg/dL should have minimized inadvertent inclusion of dogs with CKD in the healthy dog group. Future research should explore the value of acid suppression in dogs with more advanced disease (Stage IV) as well as in dogs with severe acute injury as opposed to chronic disease. Because we were concerned about the effect of placement of restrictions on feeding on our enrollment of dogs with CKD, we did not control for time, frequency, or amount of feeding although food has not been demonstrated to have a major effect on gastric pH when the capsule is adhered to the gastric mucosa rather than orally administered.^23^

In conclusion, our results suggest that dogs with CKD do not have significant gastric hyperacidity or hypergastrinemia compared to age‐matched healthy dogs. These results support current ACVIM consensus guidelines to avoid prophylactic use of acid suppressants dogs with CKD in the absence of indications of GI bleeding.^27^

## CONFLICT OF INTEREST DECLARATION

Shelly Vaden serves as Associate Editor for the Journal of Veterinary Internal Medicine. She was not involved in review of this manuscript. Jonathan Lidbury, Emily Gould, Joerg Steiner and M. Katherine Tolbert are employed at the Texas A&M gastrointestinal laboratory that performed the assays on a pay for service basis. No other authors declare a conflict of interest.

## OFF‐LABEL ANTIMICROBIAL DECLARATION

Authors declare no off‐label use of antimicrobials.

## INSTITUTIONAL ANIMAL CARE AND USE COMMITTEE (IACUC) OR OTHER APPROVAL DECLARATION

Approved by the IACUC at North Carolina State University, 19‐552 and the IACUC at Texas A&M University, 2020‐0067.

## HUMAN ETHICS APPROVAL DECLARATION

Authors declare human ethics approval was not needed for this study.
